# Low-temperature fabrication of layered self-organized Ge clusters by RF-sputtering

**DOI:** 10.1186/1556-276X-6-341

**Published:** 2011-04-14

**Authors:** Sara RC Pinto, Anabela G Rolo, Maja Buljan, Adil Chahboun, Sigrid Bernstorff, Nuno P Barradas, Eduardo Alves, Reza J Kashtiban, Ursel Bangert, Maria JM Gomes

**Affiliations:** 1 Physics Department, University of Minho, 4710-057 Braga, Portugal; 2 Rudjer Boskovic Institute, Bijenicka 54, 10000 Zagreb, Croatia; 3 LPS, Physics Department, Faculty of Sciences, BP 1796, Fès, Morocco; 4 Sincrotrone Trieste, 34012 Basovizza, Italy; 5 ITN, Ion Beam Laboratory, EN10, 2686-953 Sacavém, Portugal; 6 Nanostructured Materials Research Group, School of Materials, The University of Manchester, P.O. Box 88, Manchester, M1 7HS, UK

## Abstract

In this article, we present an investigation of (Ge + SiO_2_)/SiO_2 _multilayers deposited by magnetron sputtering and subsequently annealed at different temperatures. The structural properties were investigated by transmission electron microscopy, grazing incidence small angles X-ray scattering, Rutherford backscattering spectrometry, Raman, and X-ray photoelectron spectroscopies. We show a formation of self-assembled Ge clusters during the deposition at 250°C. The clusters are ordered in a three-dimensional lattice, and they have very small sizes (about 3 nm) and narrow size distribution. The crystallization of the clusters was achieved at annealing temperature of 700°C.

## Introduction

Semiconductor nanocrystals (NCs) have shown a big potential for application in flash memory devices [1]. Most quantum dot (QD) flash memory research studies have used Si NCs in floating gate. However, several groups have proposed systems using Ge dots [2] instead of Si dots. The band gap of Ge provides both a higher confinement barrier for retention mode and a smaller barrier for program and erase mode. This makes Ge dots a strong candidate for floating gates.

However, the fabrication of Ge dots on insulators is much more difficult to obtain than Si dots because of the low evaporation temperature of Ge and the difference in surface energy with respect to the oxide. Si_1-__*x*_Ge_*x *_can offer an intermediate solution to this issue. In fact, embedding silicon or silicon germanium (SiGe) dots in an insulator structure has been proposed for non-volatile memory devices [3-6]. Magnetron sputtering has been proven to be a useful, cheap, and easy technique with less energy consuming, for the fabrication of Si, Ge, and Si_1-__*x*_Ge_*x *_NCs embedded in SiO_2 _films [7,8].

The most challenging part in the production of nanoclusters for potential applications is the control over their size and arrangement properties. Earlier studies have reported layered Ge NCs produced at temperatures of 500°C and higher [9,10]. However, the nanoclusters formed were not regularly ordered. Recently, it has been reported of a possibility to grow self-assembled NCs in amorphous silica matrix [11,12]. However, the ordering was only found for a single deposition temperature, and it was performed only for Ge nanoclusters. The control of ordering of the particles is important because the spatial regularity implies narrowing of the QDs size distribution, which is very important for the collective behavior effects and consequently for potential applications of the system.

The complete crystallization of the NCs was achieved at temperatures of 800°C and higher [8,13,14]. In this article, we report the formation of self-assembled Ge nanoclusters by the magnetron sputtering technique at quite a low deposition temperature of 250°C. The nanoclusters formed are very small in size (about 3 nm), and well ordered in a three-dimensional FCC-like nanocluster lattice. The parameters of the nanocluster lattice formed are precisely determined using grazing incidence small angle X-ray scattering (GISAXS) and high-resolution transmission electron microscopy (HRTEM) techniques, while their crystalline quality and chemical composition are examined using Raman spectroscopy and X-ray photoelectron spectroscopy (XPS). The mutual distances of the nanoclusters are found to be very small (distance of about 3 nm between the nanocluster edges), while their size distribution is found to be very narrow. These properties make this material very suitable for different nano-based applications.

## Experimental

SiO_2_/Si_1-__*x*_Ge_*x *_+ SiO_2_/SiO_2 _multilayers films containing 20 bi-layers were prepared on Si (100) substrates using RF magnetron co-sputtering machine Alcatel SCM650. The structures were grown using a composite target, a SiO_2 _(99.99%) plate partially covered by polycrystalline chips of Si and Ge, and a second target of pure SiO_2_. The surface ratio of the Si and Ge pieces in the SiO_2 _target was 2:1. Before sputtering, a pressure of at least 1 × 10^-6 ^mbar was reached inside the chamber. Substrate and targets were subjected to *in situ *argon plasma treatment to clean the surfaces and remove any impurities. The layers were grown at 250°C, and the argon pressures were 1 × 10^-2 ^and 1 × 10^-3 ^mbar, for the pure target and the composite target, respectively. The thickness of both types of layers was controlled by the deposition time. The deposition rates were found to be 7.4 and 7.8 nm/min, for SiO_2 _and SiGe + SiO_2 _layers, respectively. The thicknesses of SiGe + SiO_2 _and SiO_2 _layers are 2 and 5 nm, respectively. A top SiO_2 _layer was deposited to prevent the diffusion of Ge atoms out of the surface. The samples were subsequently thermally annealed at temperatures between 700 and 1000°C, in N_2 _atmosphere for 1 h.

Rutherford backscattering spectrometry (RBS) measurements were performed with a 2-MeV ^4^He^+ ^ion beam impinging on the target at grazing angles of 78°, 80°, and 82° to obtain sufficiently high depth resolution to separate the signals arising from the different layers, and to detect and investigate possible compositional changes.

Conventional TEM and high-resolution TEM images were acquired with a Tecnai F30 FEG-TEM microscope operating at 300 kV. TEM cross-sectional samples were produced by mechanical polishing followed by ion beam milling to have sufficiently large electron transparent areas. GISAXS measurements were performed at the SAXS beamline of the Elettra synchrotron, using monochromatic radiation with wavelength 0.154 nm, and several grazing incidence angles slightly above the critical angle of total external reflection. The incidence direction of the X-ray radiation was along the *x *axis, perpendicular to the detector (*y*-*z*) plane. Data were measured by a two-dimensional (1024 × 1024 pixel) CCD detector, with a sample-detector distance of approx. 1.72 m. A thin Al-stripe (beam stopper) was inserted in front of the 2D detector to attenuate the very intense specular beam (reflected beam, Yoneda peak, etc.) and thus avoid the overflow of the detector, and increase the sensitivity for scattered signal outside the specular plane. Raman scattering spectra were recorded using a Jobin-Yvon T64000 system with an optical microanalysis system and a CCD detector, in the backscattering geometry. These measurements were performed at room temperature using the 488 nm line of an argon ion laser. The laser beam was focused on the sample surface with a beam spot size of 1 μm and a power of 0.2 mW to avoid the heating of the sample. XPS were measured using a Thermo Scientific K-Alpha ESCA instrument equipped with aluminum Ka1.2 monochromatized radiation at 1486.6 eV X-ray source.

## Results and discussion

**Figure 1:**
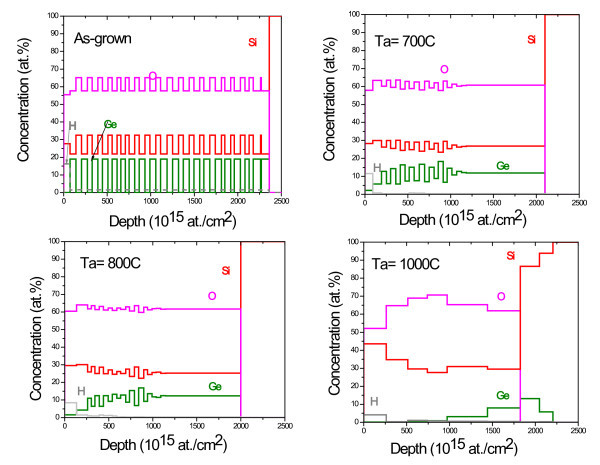
**Depth profiles of different elements (Si, O, and Ge) obtained from fits of measured RBS, for the as-grown and annealed films**.

RBS technique was applied to examine the layer structure of the as-grown multilayers. Figure 1 shows the depth profiles of the as grown and annealed films obtained from the fits [15] of the measured RBS intensity distributions. The results show a well-organized layer structure of the as-grown film (Figure 1a), with the layer thickness as expected from the growth conditions. After annealing at 700°C (Figure 1b), the samples still retain a layered structure, but for temperatures of 800°C or higher, a clear diffusion of Ge and a destruction of the multilayers structure are observed (Figure 1c,d). At 1000°C, only a small amount of Ge remains at the interface.

**Figure 2:**
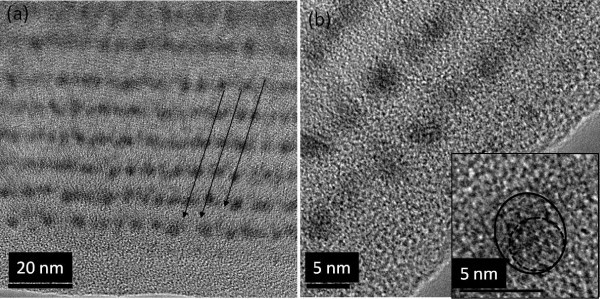
**HRTEM cross-sectional images of the as-deposited multilayer, depicted in various magnifications**. The regularity in the cluster positions is indicated by arrows. In some clusters (inset) crystallization of the deposited material is visible.

HRTEM was employed to explore the structure of the as-grown multilayers. Figure 2 shows a bright-field cross-sectional TEM image of the as-deposited multilayer sample, with different magnifications. In Figure 2a, dark dots are seen on the oxide matrix corresponding to the clusters formed, due to their higher material density. As a result of the two-dimensional projection of a three-dimensional sample, some of the layers appear to be continuous. The image with the higher magnification (Figure 2b) shows that the clusters are well separated and nearly spherical in shape. Some regularity in the nanocluster positions may be noticed (Figure 2a), but spatial correlations are much better visible in the reciprocal space, which will be shown later. Some of the as-grown clusters show a crystalline phase as illustrated in the inset of Figure 2b. This demonstrates that the as-grown sample at 250°C already contained some crystalline particles. However, more HRTEM observations are under progress to shed light on the nature (crystalline/amorphous) of the nanoparticles. The average size of particles found by HRTEM images was approximately 3 nm.

**Figure 3:**
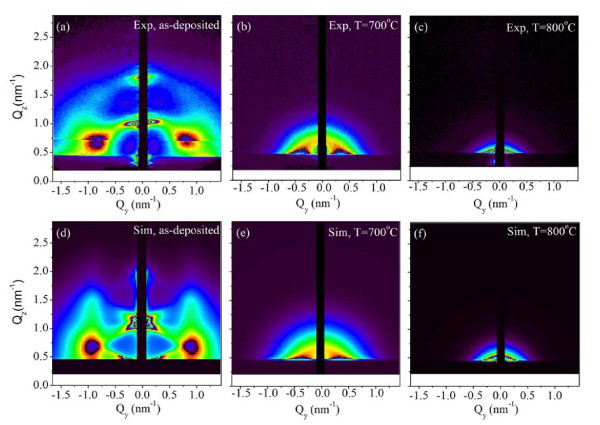
**2D GISAXS maps. **2D GISAXS maps. of (a) as deposited film (b) film annealed at 700°C, and (c) film annealed at 800°C. The second row shows the corresponding simulated GISAXS maps.

**Figure 4:**
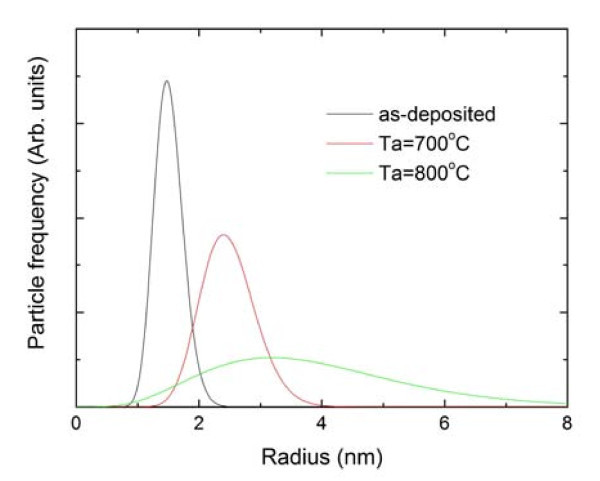
**Size distribution of the NCs obtained by the GISAXS analysis**.

GISAXS technique was applied to study the clusters' size and their arrangement properties. It gives data from a much larger sample volume compared to the TEM technique. Furthermore, the data are provided in the reciprocal space, so possible spatial correlations would appear as extra diffraction (Bragg) spots, well visible in GISAXS maps. GISAXS maps of the as-deposited and of the annealed multilayers with the corresponding simulations are shown in Figure 3. In the GISAXS map of the as-deposited film, strong Bragg spots are visible. They appear because of the existence of a 3D correlation in the cluster positions [11]. Similar to the 3D clusters reported in [11], the clusters are ordered in a distorted FCC-like lattice defined by primitive vectors ***a***_1,2,3_. Vectors ***a***_1,2 _are in the plane parallel to the substrate surface, and they form a distorted 2D hexagonal lattice. The vertical component of ***a***_3 _equals the multilayer period *T*. The regular ordering appears in domains which are randomly oriented with respect to the normal to the multilayer surface. As is explained in [11], such regular ordering is a result of interplay of diffusion-mediated nucleation and surface morphology effects. The most important point is that nanoclusters in each new layer nucleate within the minima of the existing surface, while the positions of minima are correlated to the positions of the nanoclusters in the layer underneath. The experimentally measured GISAXS map was fitted to the model described in [11] to obtain the cluster size and arrangement parameters. The results of the analysis give the following parameters for the formed nanoclusters lattice: spacing of clusters within the layers, |*a*_1_| = |*a*_1_| = 6.5 ± 0.2 nm, and the multilayer period *T *= 6.9 ± 0.1 nm, in agreement with the HRTEM results. The root mean square deviations of the clusters positions from the ideal ones are given by disorder parameters σ_L _and σ_V _describing deviations in directions parallel and perpendicular to the multilayer surface, respectively. These values are also found by GISAXS fit: σ_L _= 3.4 ± 0.2 nm and σ_V _= 0.5 ± 0.1 nm. The size distribution shown in Figure 4 is found to be very narrow for the as-deposited multilayer. Narrowing of the size distribution is a consequence of the regular ordering of the QDs [12].

In the GISAXS map of the film annealed at 700°C, a rearrangement of the Bragg spots' positions is visible. From the new arrangement, it follows that the clusters are not any more correlated in the vertical direction, while the correlation of lateral clusters still exists. The results of the numerical analysis show formation of NCs which are larger than in as-deposited multilayer (*R *= 2.5 ± 0.3 nm), with larger mutual distance (*L *= 17.8 ± 0.3 nm) and significantly larger vertical disorder parameter (σ_V _= 1.6 ± 0.1 nm). The in-layer disorder is also larger than for the as-deposited case (σ_L _= 9.1 ± 0.1 nm), but the separation *L *is also larger. Growth of QDs during the annealing treatment causes the destruction of the vertical dot correlation. Initially regularly ordered QDs coalesce, thereby changing their lateral positions. The size distribution is still relatively narrow, but broader than in the as-deposited film case. Annealing at 800°C causes a further growth of QDs (*R *= 3.8 ± 0.5 nm), and a further decrease of the regularity in the QD positions. For this film (Figure 3c), no Bragg spots are visible in the GISAXS intensity distribution. The size distribution, shown in Figure 4, is found to be very broad in this film.

**Figure 5:**
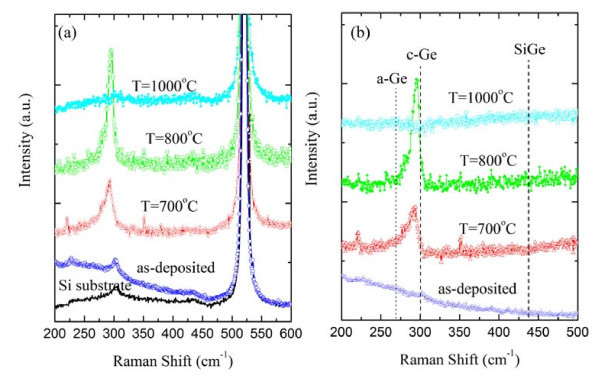
**Raman spectra of as-deposited and annealed multilayers**. (a) Raman spectra of the as-deposited and annealed multilayers at temperatures indicated in the figure. The spectra are normalized to the intensity of Si-substrate peak at 520 cm^-1^. (b) The same spectra after the subtraction of Si substrate contribution. Dashed lines show the positions of peaks of amorphous Ge (a-Ge), crystalline Ge (c-Ge), and Si-Ge vibrational modes.

We employed Raman spectroscopy which is a very effective tool to study the crystalline structure and the stoichiometry of the nanoparticles. Figure 5a shows the Raman spectra of the as-deposited, annealed multilayers and Si substrate, and Figure 5b shows the same spectra after the subtraction of Si substrate contribution. The as-grown multilayer shows a broad band near to 270 cm^-1^, which is characteristic of amorphous Ge [16]. The samples annealed at 700 and 800°C show strong peaks at 292 and 295 cm^-1^, respectively. These peaks show existence of crystalline Ge (c-Ge) nanoparticles in the film. The peaks are slightly red-shifted and asymmetrically broadened with respect to the Ge bulk peak (300.4 cm^-1^) because of the phonon confinement in the nano-sized particles [17]. The shifts are in accordance with the results of GISAXS analysis showing formation of Ge clusters with radii of 2.5 and 3.8 nm for the films annealed at 700 and 800°C, respectively. A small peak coming from the Si substrate exists near to 304 cm^-1^; however, for the annealed samples, this peak is associated to Ge NCs. The samples annealed at 1000°C do not show any Raman peak because of NCs, and only the Raman signal arising from the silicon substrate is observed. This absence of Raman peak can be attributed to the loss of Ge atoms during the annealing. We have already observed a total loss of Ge atoms from the Al_2_O_3 _film during thermal treatments, because of the volatilization of Ge mono-oxide (GeO) [18]. In the present case, the loss of Ge is partial, since RBS spectra of the samples reveal the presence of Ge atoms in the layers near the interface film-substrate. The lack of the presence of for any Raman feature can be interpreted as a consequence of the decrease in the amount of material inside the scattering volume. Rodriguez et al. [14] observed a similar behavior, and concluded that, after a certain annealing temperature, the compositional changes due to the out-diffusion of Ge from the crystallized nanoparticles and the associated reduction of the scattering volume cause the NCs to fall below the detection limit of the Raman setup, thus accounting for the disappearance of the Raman signal. The observed absence of Si-Ge and Si-Si Raman peaks for the annealed samples could be explained by the low amount of Si used during the growth and/or a loss of Si atoms during the thermal treatments, which can oxidize and form SiO_2_.

**Figure 6:**
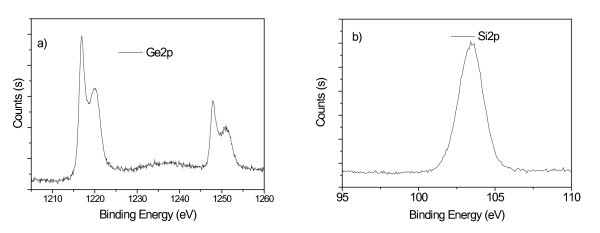
**XPS spectra**. XPS Ge 2p (a) and Si 2p (b) for the as-grown multilayer.

In our attempt to clarify the chemical composition of the nanoparticles, we have performed XPS analyses of the as-grown multilayer. Peaks relative to Ge 2p and Si 2p are shown in Figure 6a,b, respectively. The signal due to Ge exhibits a double peak features because of pure Ge and GeO*x *states. From the XPS data only Ge, GeO, and SiO*x *were detected. No Si-Ge formation was observed in agreement with the Raman results.

Contrary to the general tendency observed in the literature concerning the growth of NCs, we have shown the possibility to grow the self-assembled nanoclusters at low temperature (250°C). Low-cost process will be explored further to obtain well-separated crystalline NCs.

## Conclusions

In this study, we have shown formation of self-organized Ge nanoclusters at low temperature (250°C) in amorphous silica matrix by the magnetron sputtering technique. The size distribution of the clusters formed is found to be very narrow because of the self-ordering growth. The annealing of those films caused the formation of crystalline Ge clusters with larger sizes. Furthermore, the regular spatial arrangement of clusters has undergone changes by the annealing treatment. RBS results show that annealing at 800 and 1000°C promote the out-diffusion from the surface of Ge atoms.

## Abbreviations

GISAXS: grazing incidence small angle X-ray scattering; HRTEM: high-resolution transmission electron microscopy; NCs: nanocrystals; QD: quantum dot; RBS: Rutherford backscattering spectrometry; XPS: X-ray photoelectron spectroscopy.

## Competing interests

The authors declare that they have no competing interests.

## Authors' contributions

SRCP carried out the sample growth experiment and characterisation analysis and drafted the manuscript.AGR participated in the design of the study, carried out the Raman experiments, and characterisation analysis, as well as drafted the manuscript. MB participated in the design of the study, carried out the GISAXS experiments, performed the statistical analysis, as well as drafted the manuscript. AC participated in the design of the study and revised the manuscript. SB carried out the GISAXS experiments, performed the statistical analysis, and revised the manuscript. NPB and EA carried out the RBS experiments, performed the statistical analysis, and revised the manuscript. RJK and UB carried out the HRTEM experiments, and revised the manuscript. MJMG participated in the coordination of study. All authors read and approved the final manuscript.
